# Circular RNA expression profiles and features in human tissues: a study using RNA-seq data

**DOI:** 10.1186/s12864-017-4029-3

**Published:** 2017-10-03

**Authors:** Tianyi Xu, Jing Wu, Ping Han, Zhongming Zhao, Xiaofeng Song

**Affiliations:** 1 Department of Biomedical Engineering, Nanjing University of Aeronautics and Astronautics, No. 29 Jiangjun Avenue, Nanjing, Jiangsu 211106 China; 2 Department of Gynecology and Obstetrics, The First Affiliated Hospital, Nanjing Medical University, Nanjing, Jiangsu 210029 China; 3 Center for Precision Health, School of Biomedical Informatics, The University of Texas Health Science Center at Houston, 7000 Fannin St., Suite 820, Houston, TX 77030 USA; 4 Human Genetics Center, School of Public Health, The University of Texas Health Science Center at Houston, Houston, TX 77030 USA

**Keywords:** Circular RNA, Expression profile, microRNA ‘sponge’, Tissue specificity, Regulatory network, Mammary gland

## Abstract

**Background:**

Circular RNA (circRNA) is one type of noncoding RNA that forms a covalently closed continuous loop. Similar to long noncoding RNA (lncRNA), circRNA can act as microRNA (miRNA) ‘sponges’ to regulate gene expression, and its abnormal expression is related to diseases such as atherosclerosis, nervous system disorders and cancer. So far, there have been no systematic studies on circRNA abundance and expression profiles in human adult and fetal tissues.

**Results:**

We explored circRNA expression profiles using RNA-seq data for six adult and fetal normal tissues (colon, heart, kidney, liver, lung, and stomach) and four gland normal tissues (adrenal gland, mammary gland, pancreas, and thyroid gland). A total of 8120, 25,933 and 14,433 circRNAs were detected by at least two supporting junction reads in adult, fetal and gland tissues, respectively. Among them, 3092, 14,241 and 6879 circRNAs were novel when compared to the published results. In each adult tissue type, we found at least 1000 circRNAs, among which 36.97–50.04% were tissue-specific. We reported 33 circRNAs that were ubiquitously expressed in all the adult tissues we examined. To further explore the potential “housekeeping” function of these circRNAs, we constructed a circRNA-miRNA-mRNA regulatory network containing 17 circRNAs, 22 miRNAs and 90 mRNAs. Furthermore, we found that both the abundance and the relative expression level of circRNAs were higher in fetal tissue than adult tissue. The number of circRNAs in gland tissues, especially in mammary gland (9665 circRNA candidates), was higher than that of other adult tissues (1160–3777).

**Conclusions:**

We systematically investigated circRNA expression in a variety of human adult and fetal tissues. Our observation of different expression level of circRNAs in adult and fetal tissues suggested that circRNAs might play their role in a tissue-specific and development-specific fashion. Analysis of circRNA-miRNA-mRNA network provided potential targets of circRNAs. High expression level of circRNAs in mammary gland might be attributed to the rich innervation.

**Electronic supplementary material:**

The online version of this article (doi:10.1186/s12864-017-4029-3) contains supplementary material, which is available to authorized users.

## Background

Cellular RNAs can be separated into two categories: coding and noncoding. There are many noncoding RNAs, such as rRNA, tRNA, snRNA, snoRNA, long noncoding RNA (lncRNA) and microRNA (miRNA). Numerous studies have reported that noncoding RNAs play important regulatory roles in biological processes. Among the noncoding RNA types, circular RNA (circRNA) has recently been found with promising regulatory roles in cellular systems, though its expression level is typically low. Compared with linear RNA, circRNA exhibits remarkable characteristics of undergoing non-canonical splicing without 5′-cap structure or 3′-polyadenylated tail; accordingly, Jeck et al. proposed two models of circRNAs formation [1]. Model 1 is termed ‘lariat-driven circularization’ or ‘exon skipping’, while model 2 is termed ‘intron-pairing-driven circularization’ or ‘direct backsplicing’. This class of noncoding RNAs is characterized by a stable structure in cells that is typically not affected by RNase R treatment [2, 3]. CircRNAs were first time reported in viroid in 1976 [4, 5]. With the rapid advances in next generation sequencing technologies, many circRNAs, along with other noncoding RNAs, have been identified in mammalian transcriptomes [6–13].

CircRNA represents a large class of noncoding RNAs. Recent studies indicated that circRNAs have regulatory potency to mediate gene expression. It was recently reported that circRNAs can function as miRNA’s ‘sponges’ that naturally sequester and competitively suppress miRNA activity [14–16]. For example, two circRNAs, ciRS-7/CDR1as (circular RNA sponge for miR-7 or CDR1 antisense) and Sry, have been shown to bind miRNAs without being degraded [14]. CiRS-7/CDR1as strongly suppresses miR-7 activity, which results in an increased expression of miR-7’s target genes. The sex-determining region Y (Sry) has been proven to serve as a miR-138 sponge [17]. Liu et al. discovered that circRNAs-CER regulated MMP13 expression by functioning as a competing endogenous RNA (ceRNA) [18]. In addition, circRNAs may act as ‘scaffolding’ for RNA-binding proteins (RBPs), like AGO proteins and Pol II [14]; therefore, it could regulate the biological process of associated RBPs in cell. Other studies also demonstrated that circRNAs have been involved in the development of various diseases, including atherosclerosis, nervous system disorders, and cancer [18–20]. Guarnerio et al. showed that tumors harboring chromosomal translocation also harbor circRNAs derived from the rearranged genome: aberrant fusion-circRNA (f-circRNA). They further demonstrated that those f-circRNAs could be functionally relevant in promoting tumorigenesis, implicating their diagnostic and therapeutic potentials [21]. To investigate whether circRNAs could play critical roles in other diseases, it is important to understand how circRNAs are expressed in different human tissues and the differential expression of the circRNAs in disease samples versus normal samples. So far, there has been little information available in literatures about circRNA expression profiles in different human tissues, especially the features in adult versus fetal tissues. Such knowledge is important for the above investigation.

In this study, we performed a systematic examination of circRNAs in a variety of human normal tissues. We reported thousands of circRNAs in colon, kidney, heart, liver, lung and stomach tissues, respectively. Importantly, 33 circRNAs were identified to be pervasively expressed in all the human normal tissues and in both adult samples and fetal samples. These “housekeeping-like” circRNAs might have high conservation and play basic function in normal tissues. We also found 25,933 circRNAs in fetal samples, and 1094, 1991, 1664, 939, 1701, 638 circRNAs that had significantly higher expression in fetal colon, heart, kidney, liver, lung, and stomach tissues than their corresponding adult tissues. Our results suggested potentially critical roles of circular RNAs in human development. We also analyzed four types of gland normal tissues (adrenal gland, mammary gland, pancreas, and thyroid gland) in human body. The expression of circRNAs in mammary gland was higher than that in three other gland tissues. This result suggested that high level of circRNA expression in mammary gland might be attributed to the rich innervation.

## Materials and methods

### Datasets

We collected 36 publicly available datasets, including 27 rRNA-depleted total RNA-seq samples from six normal tissues (colon, heart, kidney, liver, lung, and stomach) and 9 rRNA-depleted total RNA-seq samples from four normal gland tissues (adrenal gland, mammary gland, pancreas, and thyroid gland). Here, rRNA denotes ribosomal RNA. There were 5 individual samples for adult stomach tissue, 3 individual samples for adult mammary gland, and 2 individual samples for each adult and fetal tissue. All these datasets were retrieved from NCBI [22] and ENCODE [23], and all were paired-end RNA-seq data (Additional file 1: Table S1).

### Identification and quantification of human circRNAs

For each sample, the cleaned RNA-seq reads were first mapped to the human reference genome (GRCh37/hg19, UCSC Genome Browser [24]) by TopHat2 [25], a software tool that is capable of detecting the canonical splicing event. Then, the unmapped reads of each sample in the TopHat2 results were used to identify the circRNAs by UROBORUS pipeline [26]. Briefly, the unmapped reads were extracted to 20-bp anchors from head ends and tail ends. This short 20-bp paired-end seed was aligned to the human reference genome (hg19) with a maximum of 2 bp mismatches using TopHat2 with the default parameters. This process generated two sets of reads spanning the spliced site: balanced mapped junction (BMJ) reads and unbalanced mapped junction (UMJ) reads. BMJ reads are represented as reads aligned to the joining region of two back-spliced exons with minimum 20 bp of overhang at any end of the reads; UMJ reads are represented as reads aligned to the joining region of two back-spliced exons with less than 20 bp of overhang at one end of the reads. The UROBORUS pipeline designed special algorithm to deal with them, so the reads supporting circRNAs could be detected.

To evaluate the relative expression of circRNAs in different samples and different normal tissues, we counted all the reads mapped to the human reference genome (hg 19) and normalized the number of circular reads to per million number of reads mapped to the genome, abbreviated as RPM.

### Evaluate the corresponding parental mRNAs’ expression level of circRNAs

To evaluate the corresponding parental mRNAs’ expression level of circRNAs, we used software Cufflinks [27] (version 2.2.1) to process the accepted_hits.bam file from TopHat2 results; this file contained all the reads mapped to the human reference genome. Consequently, we estimated the relative expression level of the parental mRNA in reads per kilobase per million mapped reads (RPKM).

### Prediction of miRNAs related to circRNAs

To predict the relationship between the miRNAs and the identified circRNAs, the sequences and annotation of published miRNAs were obtained from miRBase database [28]. We selected the high confidence miRNAs in humans. Then, miRanda pipeline [29] was used to predict the circRNA-miRNA interaction network. We set the match score higher than 170 and the minimum free energy less than −30 to improve the reliability of our prediction.

### Functional enrichment analysis using gene ontology (GO) terms

The potential target genes in circRNA-miRNA-mRNA interaction network of adult and gland normal tissue samples were used for the functional enrichment analysis. A total of 33 and 56 circRNAs in adult tissues and gland samples, respectively, were selected to construct the circRNA-miRNA-mRNA interaction network. The enrichment analysis was performed using GO biological processes and molecular function terms through the software DAVID Bioinformatics Resource v6.8. [30].

## Results and discussion

In summary, we used the RNA-seq data (total RNA with ribosomal RNA being depleted) from six adult and fetal normal tissues (colon, heart, kidney, liver, lung, and stomach) and four gland adult normal tissues (adrenal gland, mammary gland, pancreas, and thyroid gland) to identify the circRNAs using the tool UROBORUS.

### CircRNA abundance in human adult normal tissues

**Fig. 1:**
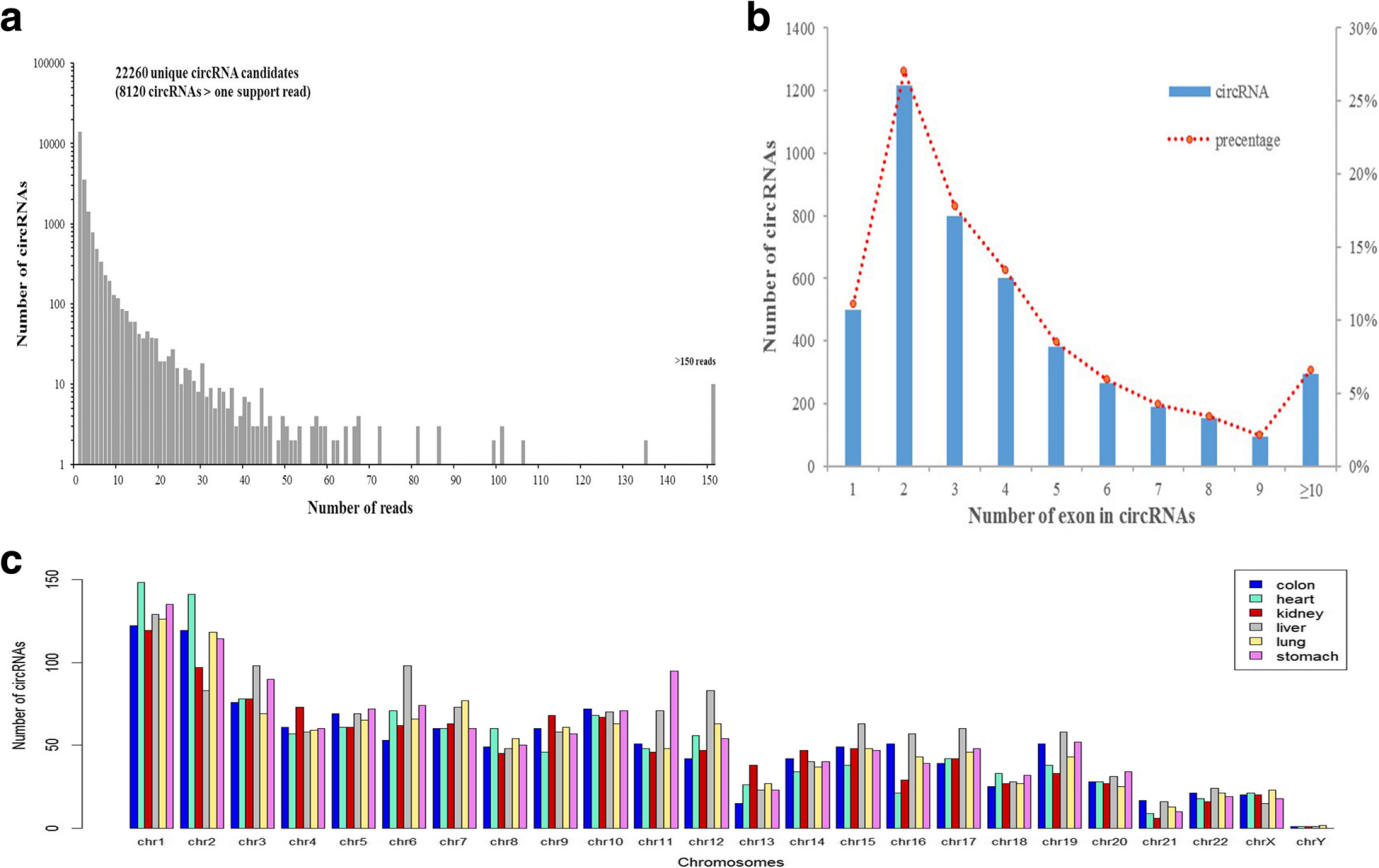
Distribution of circRNAs in human adult normal tissues. **a** Distribution of circRNAs by the number of supporting reads in human adult normal tissues. **b** Distribution of the exon number of circRNAs in human adult normal tissues. **c** Distribution of the circRNAs among human chromosomes of six adult human normal tissues

In human adult normal tissues, 22,260 unique circRNAs were identified, among which 8120 circRNA candidates were supported by at least two reads spanning a head-to-tail splice junction (Fig. 1a). When we searched the previously published circRNAs deposited in database circBase [31], we found that 9332 circRNAs have been included in the circBase while a total of 12,928 were novel circRNAs (Additional file 1: Table S2-S3). These novel circRNAs included 3092 that were supported by at least two junction reads, that is, circRNAs with high confidence. We noticed that approximately 88% circRNAs were supported by less than 10 reads. Meanwhile, there were 10 circRNA candidates supported by more than 150 reads in all adult samples. Among them, circSLC8A1 (hsa_circ_0000994) had the largest number of supporting reads (823 reads).

We then examined the number of exons for the circRNAs having at least 2 supporting junction reads. We found that majority of the circRNAs (77.74%) had less than 6 exons, indicating that only a small proportion of circRNAs consist of multiple exons by back-splicing (Fig. 1b). This result is consistent with the previous report that circRNAs biogenesis may follow two mechanisms: “direct back-splicing” and “exon skipping” [1, 32, 33]. We manually checked these identified circRNAs’ parental gene using Integrative Genomics Viewer (IGV). The parental genes for those circRNAs with two or more exons did not have corresponding exon-skipping phenomenon. However, the results showed that the derived gene of 35 circRNAs (35/495, 7.07%) had corresponding “exon skipping” transcripts; these circRNAs had only one exon. These results implied that these circRNAs with only one exon might be formed through the “exon skipping” mechanism, while the other circRNAs might be formed by “direct back-splicing” or other mechanism.

For all the adult normal tissues except stomach, the detected circRNAs were distributed on all the human chromosomes (autosomes and sex chromosomes). In stomach tissue, circRNAs were distributed on all the chromosomes but not on Y chromosome (Fig. 1c). In each tissue, our results showed that the number of circRNAs distributed on chromosome 1 was larger than any other chromosomes, while the number of circRNAs on Y chromosome was the smallest among all the chromosomes. This observation is consistent with the chromosome length – Y chromosome is the shortest and has the smallest number of genes in the human genome. In our comparison of the number of circRNAs versus chromosome length (in Megabase, Mb), we found that the circRNA density on chromosome 19 (4.69 per Mb) stood out, which had the highest density among all the chromosomes (average 2.55 per Mb). These results are interesting because the gene density of chromosome 19 is also the highest among chromosomes [34]. However, the distribution of circRNAs on autosomes and X chromosome is not uniform in each normal tissue. For example, the number of circRNAs on chromosome 6 (98) and chromosome 12 (83) in liver was higher than that of other tissues (mean values: 65.2 and 52.4). The number of circRNAs on chromosome 11 in stomach (95) was also higher than that of other tissues.

**Fig. 2:**
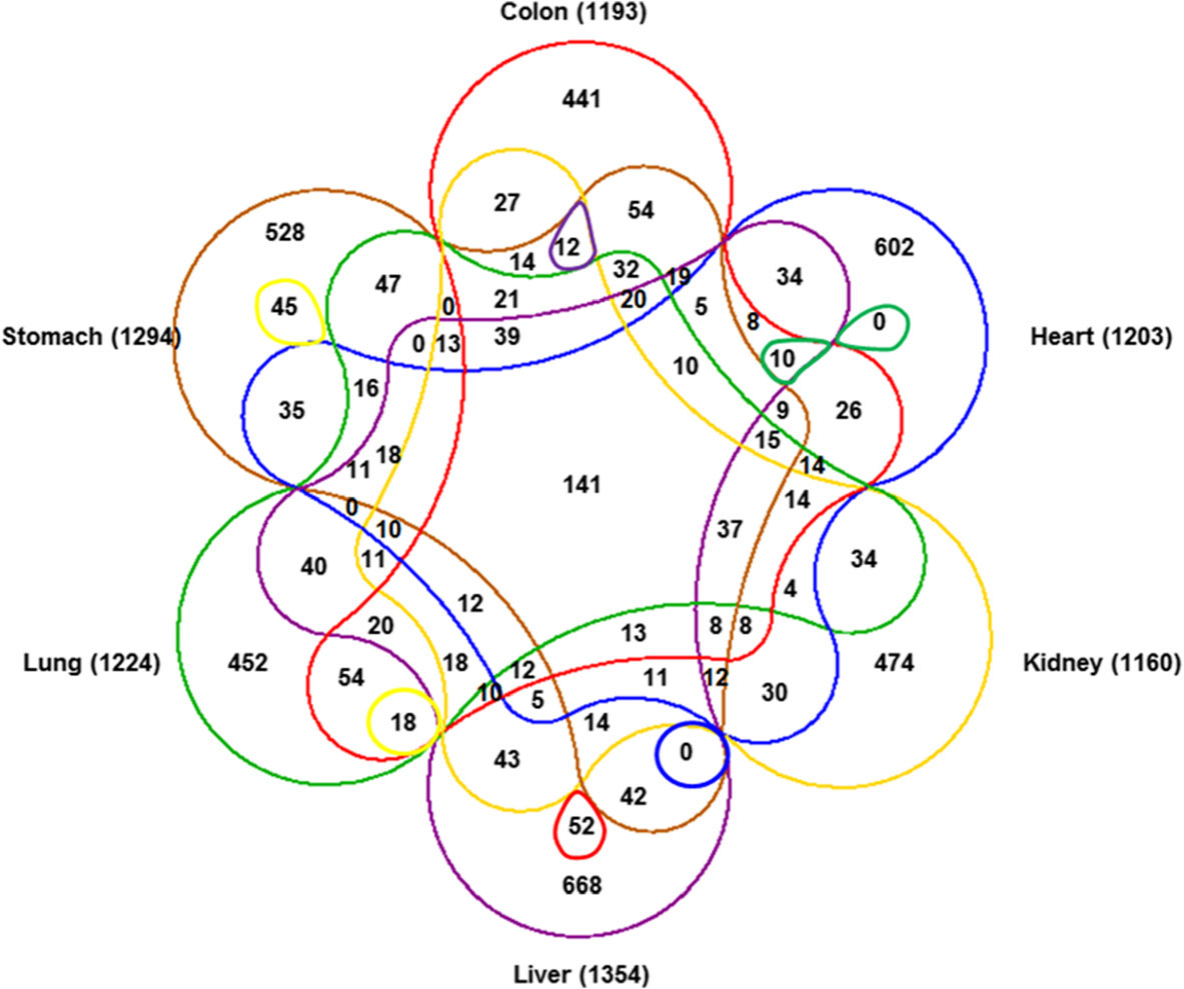
Venn diagram summarizing the circRNAs (supported by at least two reads) shared among six human adult normal tissues

More than one thousand circRNAs (with at least two supporting junction reads) were identified in each human adult normal tissue (Fig. 2). Each tissue had a good number of unique circRNAs, for example, 441 (36.97%) in colon, 602 (50.04%) in heart, 474 (40.86%) in kidney, 668 (49.34%) in liver, 452 (36.92%) in lung, and 528 (40.80%) in stomach. Moreover, there were 141 circRNAs shared in all normal tissues, even though the expression level of circRNAs was different among these tissues. The result supported that some circRNAs are expressed in all tissues (e.g. in “housekeeping-like” role). These 141 circRNAs may have same or similar function in different adult normal tissues.

Although we found that the number of circRNAs detected in each tissue type was nearly equivalent, the expression of the top ten circRNAs differed in tissue types, as illustrated in Additional file 2: Figure S1. Specifically, circHIPK3 (hsa_circ_0000284) and circUBXN7 (hsa_circ_0001380) were the top two ranked based on the expression in each tissue. The circHIPK3 expression, measured in RPM (junction reads per million mapped reads), was the highest in colon, kidney, lung and stomach tissues, and the average expression level of circSLC8A1 in heart tissue and the circZKSCAN1 (hsa_circ_0001727) in liver tissue was higher than other tissues, suggesting that the circSLC8A1 and circZKSCAN1 may respectively have special function in heart and liver than any other tissues.

**Fig. 3:**
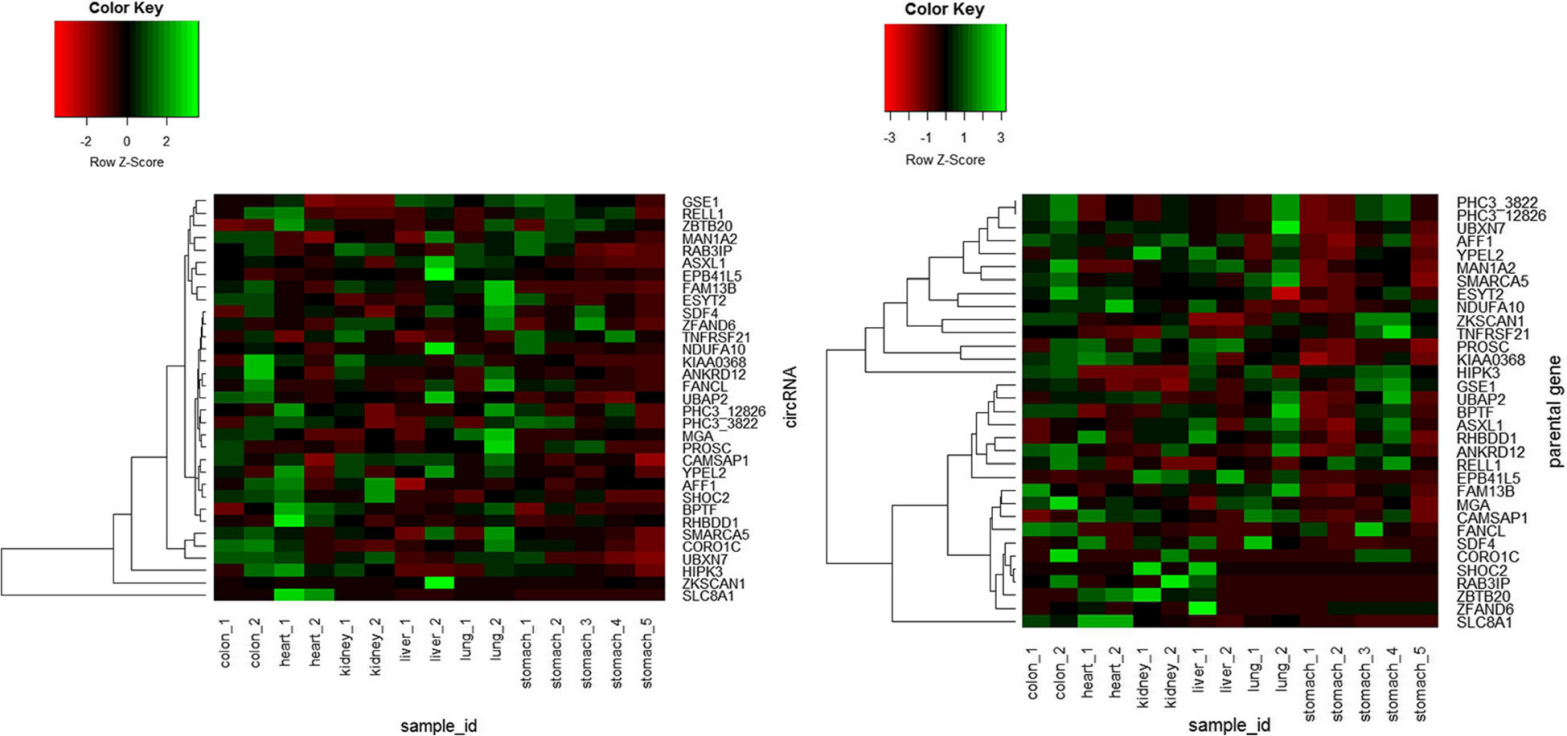
The heatmaps of the 33 shared circRNAs and their parental mRNA expression level in six human adult normal tissues

There were a total of 33 circRNAs ubiquitously expressed in each sample of all adult normal tissues (Additional file 1: Table S4-S5). Figure 3 displays the heatmap of commonly expressed circRNAs (in RPM) and their parental mRNA expression level (FPKM – fragments per kilobase of exon per million mapped reads). Of note, the expression of these circRNAs in stomach_#4 sample (average 0.04) and stomach_#5 sample (average 0.08) was lower than other tissues, and the circRNAs expressed in liver_#2 sample (average 0.18) was higher than other tissues. Moreover, these 33 circRNAs expressed in different samples of the same tissue were quite different, but the expression of the corresponding parental mRNAs in different individuals of tissue was pretty similar (Pearson correlation coefficient (PCC) = 0.80–0.95), except in lung tissue (PCC = 0.35).

**Fig. 4:**
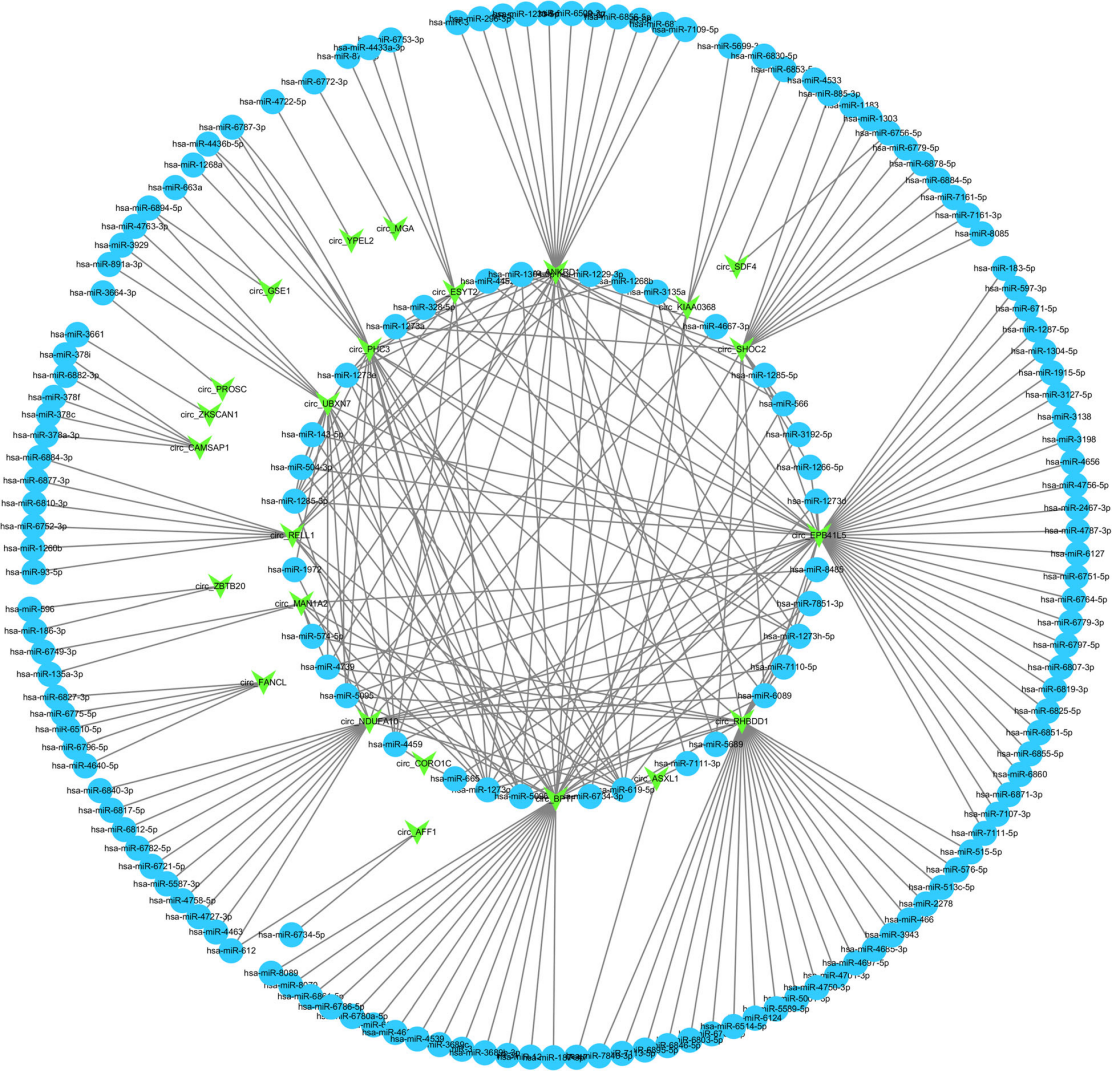
CircRNA-miRNA interaction network. The network consists of 33 circRNAs and 158 miRNAs. The *green node* represents circRNA and the *blue node* represents miRNA. Edge denotes the relationship between circRNA and miRNA

**Fig. 5:**
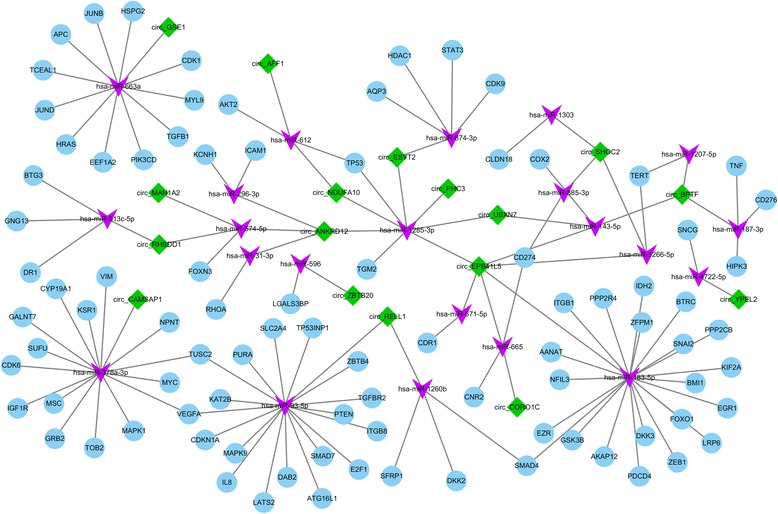
CircRNA-miRNA-mRNA interaction network. The network consists of 17 circRNAs, 22 miRNAs and 90 mRNAs. The *green node* represents circRNA, the *purple node* represents miRNA, and the *blue node* represents mRNA. Edge denotes their relationship

As shown in the results above, these 33 circRNAs exert interesting features. Considering that circRNAs can play a role as miRNA ‘sponges’ to regulate gene expression, we selected 2588 published, high confidence human miRNAs from miRBase, and used the miRanda pipeline to test which miRNA can interact with these 33 circRNAs (more details were included in Materials and methods). Most circRNAs could bind multiple miRNAs and different circRNAs might have same potential miRNA targets. Therefore, we built a circRNA-miRNA interaction network, which contains 33 circRNAs and 158 miRNAs to reflect its complex interaction and regulation (Fig. 4). Next, we employed the dataset from miRTarBase [35], which contained the experimentally validated miRNA-target interactions to predict the potential role for circRNAs in molecular regulation. Then, we used network visualization software Cytoscape (version 3.3.0) [36] to display the network, and through the network analysis, we found a total of 17 circRNAs, 22 miRNAs and 90 mRNAs in circRNA-miRNA-mRNA interaction network. In the network, each gene corresponds to a node, and 2 genes are connected by an edge, indicating a tight correlation between those genes and a potential regulatory relationship (Fig. 5).

To further explore the potential function of these 17 circRNAs in organ development or differentiation, we analyzed the GO enrichment of these 90 mRNAs-derived genes in network (Additional file 3: Figure S2). We found the genes were enriched in the regulation of cell death or apoptosis. In GO molecular function category, these circRNAs were mostly enriched in transcription activity. There is no tissue-specific function detected in the cluster. Therefore, these results might explain why these circRNAs could be shared among the tissues we examined.

### CircRNAs expression profile in human fetal normal tissues

**Fig. 6:**
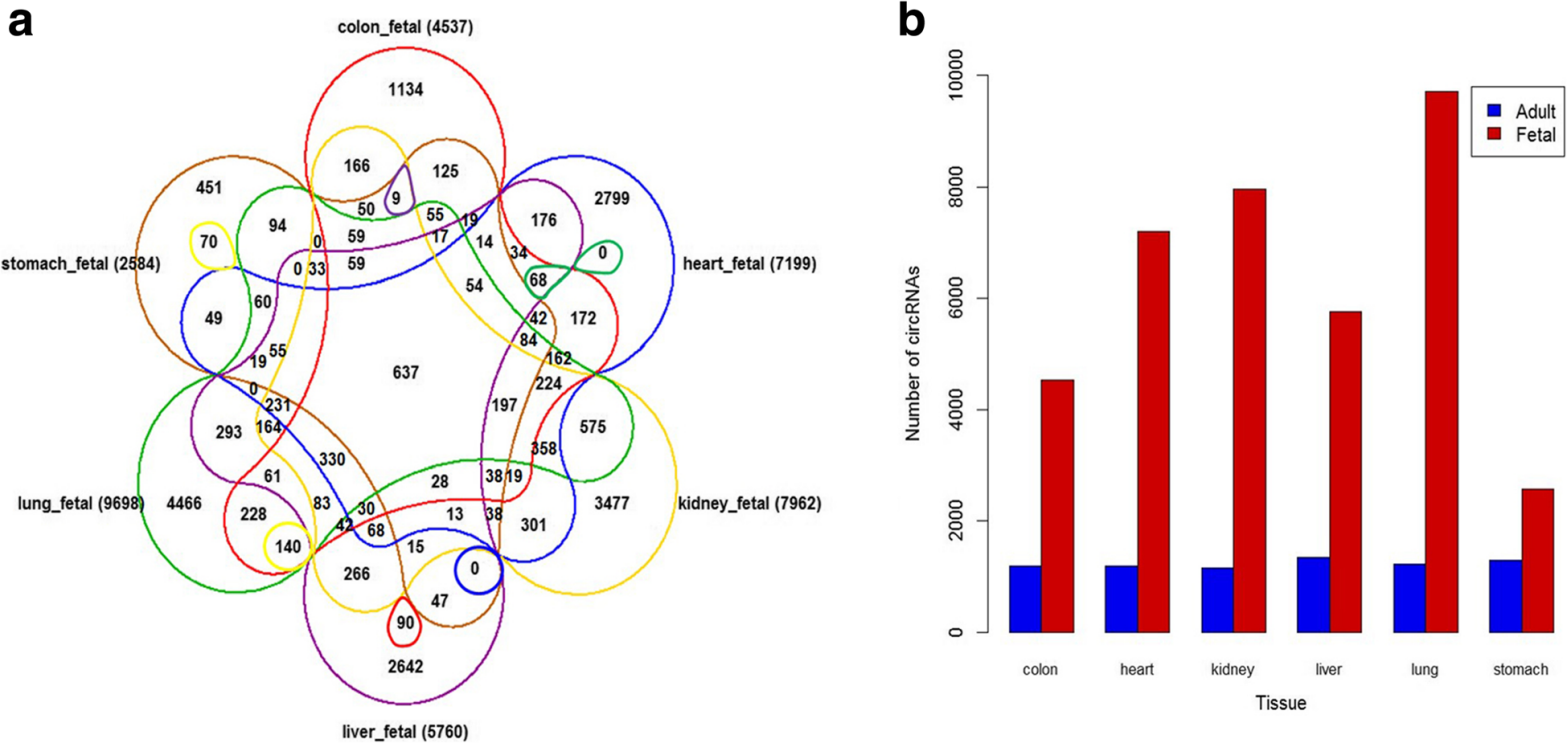
Distribution of circRNAs in human fetal normal tissues. **a** Venn diagram summarizing the circRNAs (supported by at least two reads) shared among the human fetal tissues. **b** Comparison of the number of circRNAs (supported by at least two reads) in adult and fetal tissues

In this work, we systematically analyzed 12 samples of human fetal normal tissues, including colon, heart, kidney, liver, lung, and stomach. We found a total of 47,278 circRNAs in human fetal tissues, among which 25,933 circRNAs were supported by at least two junction reads (Additional file 1: Table S6). Compared with the previously published databases obtained from circBase, 31,158 circRNAs were novel circRNA, including 14,241 circRNAs were supported by at least two reads. As shown in Fig. 6a, we identified 4537 circRNAs in colon, 7199 circRNAs in heart, 7962 circRNAs in kidney, 5760 circRNAs in liver, 9698 in lung, and 2584 circRNAs in stomach. Among these circRNAs, 637 circRNAs shared in all human fetal normal tissues. Compared to the corresponding human adult tissues, the circRNAs (average 6290) were substantially highly abundant in fetal tissues than that in adult tissues (average 1238) (Fig. 6b). For example, 9698 circRNA candidates were detected in fetal lung tissues, which is nearly an 8-fold increase to the number of circRNAs identified in adult lung tissues.

Compared with the circRNAs distributed in adult normal tissues, there were 1916 circRNAs (more than 10 supporting reads) only expressed in fetal human tissues. We analyzed the GO enrichment of potential target genes derived from the circRNA-miRNA-mRNA network. A total of 487 GO function enrichment terms, among which 56 GO terms (11.4%) are related to the development (Additional file 1: Table S7). For one example, there were 105 genes involved in GO term ‘positive regulation of developmental process’ (GO ID: 0051094), resulting in a statistically significant enrichment (Fisher’s Exact test, *p* = 2.31E-36).

Furthermore, we examined the circRNA expression and their parental mRNA expression in human fetal normal tissues. As shown in Additional file 4, 5, 6, 7, 8 and 9: Figures S3-S8, each sample had abundant high-expression specific circRNAs when compared to others, which might lead to individual specificity. We further found that there were a small number of circRNAs with high-expression, but the corresponding parental mRNA had low expression or even no expression; the results revealed that the formation mechanism of circRNAs might be different from mRNA.

The expression level of most circRNAs in each fetal tissue was generally higher than the corresponding circRNAs expressed in adult tissue of colon, liver, heart, kidney and lung, respectively. The same is true for the corresponding parental mRNA expression of each sample in different tissues. The heatmap in Additional file 9: Figure S8 showed that the circRNAs expressed in fetal stomach tissue was higher than that expressed in adult stomach tissue. However, their parental mRNA expression in adult stomach samples was higher than the circRNAs’ parental mRNA expression in fetal stomach samples.

Most of the circRNAs expression and their parental mRNA expression followed the same trend, that is, the expressed level was higher in fetal tissues than adult tissues. However, this is not always true for the samples from stomach tissue. The potential function of circRNAs in stomach tissue might be involved with different mechanism compared to other tissues. And this needs further investigation.

### CircRNAs expression in human gland normal tissues

Gland is an organ that synthesizes substances for release into the bloodstream or into cavities inside the body or its outer surface. Thus, it plays an important role in the growth and development of human body. Accordingly, we selected four kinds of gland tissues: adrenal gland, mammary gland, pancreas, thyroid gland, to explore the distribution of circRNAs in human.

**Fig. 7:**
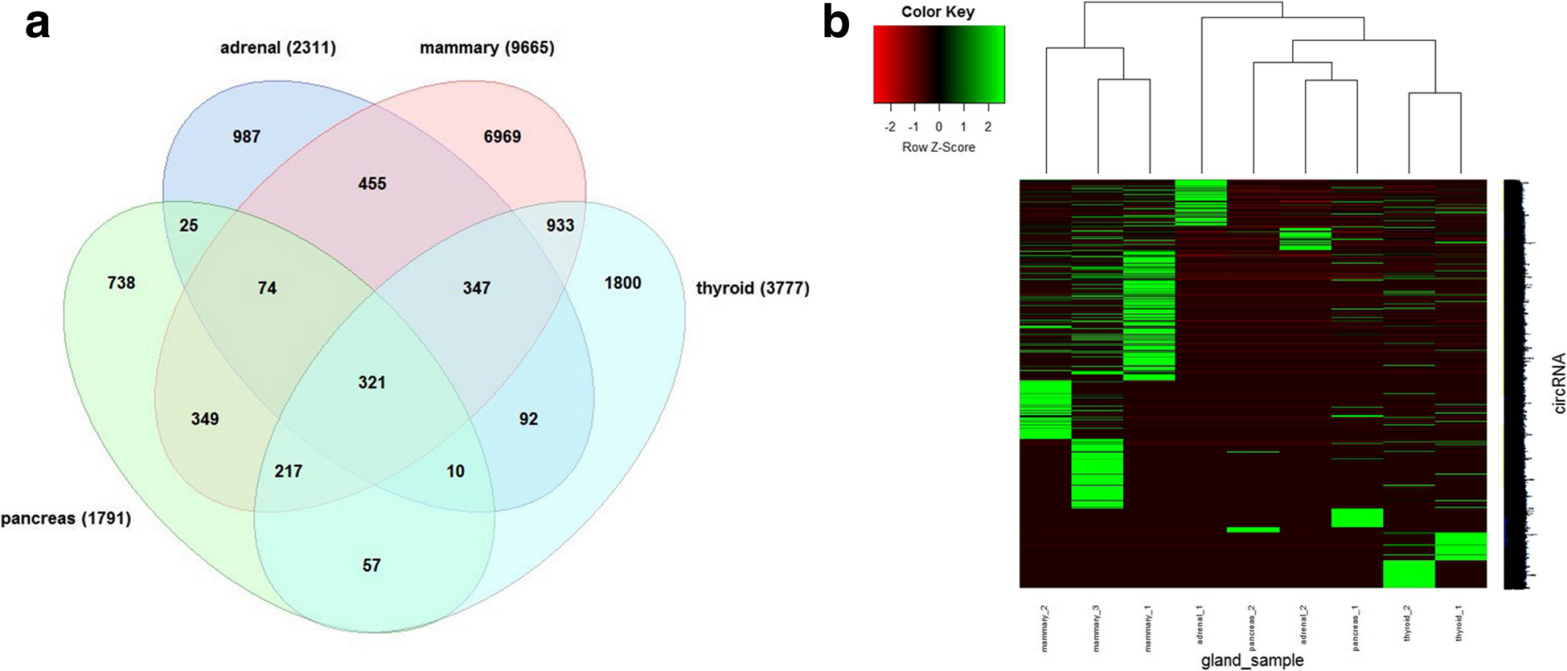
Distribution of circRNAs in adult gland normal tissues. **a** Venn diagram summarizing the circRNAs (supported by at least two reads) shared among the four human gland tissues. **b** Heatmap showing the circRNA expression in each sample of adrenal gland, mammary gland, pancreas and thyroid gland

We found a total of 23,613 circRNAs in these human gland normal tissues, and 14,433 circRNAs were supported by at least two junction reads (Additional file 1: Table S8). As the Fig. 7 illustrates, there are 2311 circRNAs in adrenal gland, 9665 circRNAs in mammary gland, 1791 circRNAs in pancreas, and 3777 circRNAs in thyroid gland. These circRNAs were identified by UROBORUS (Fig. 7a). Among these circRNAs, we found 321 circRNAs shared among the four glands. The number of circRNAs in pancreas tissues was the least among these four gland tissues, but even so, it was still more abundant than that in any human adult tissues (Fig. 2). The number of circRNA candidates recognized in mammary gland tissues was higher than almost all the human adult and fetal tissues, except for fetal lung tissue. On the other hand, the expression level of circRNAs in mammary gland was also higher than that in other gland tissues (Fig. 7b). Previous studies indicated an abundance number of circRNAs expressed in brain, due to the neuronal activity in accordance with the biogenesis mechanism of circRNAs [37]. Thus, more circRNAs expressed in mammary gland than other tissues might connect with the rich innervation of mammary gland [38]. Other factors might contribute to this abundance of circRNAs in mammary gland, and future investigation is needed.

**Fig. 8:**
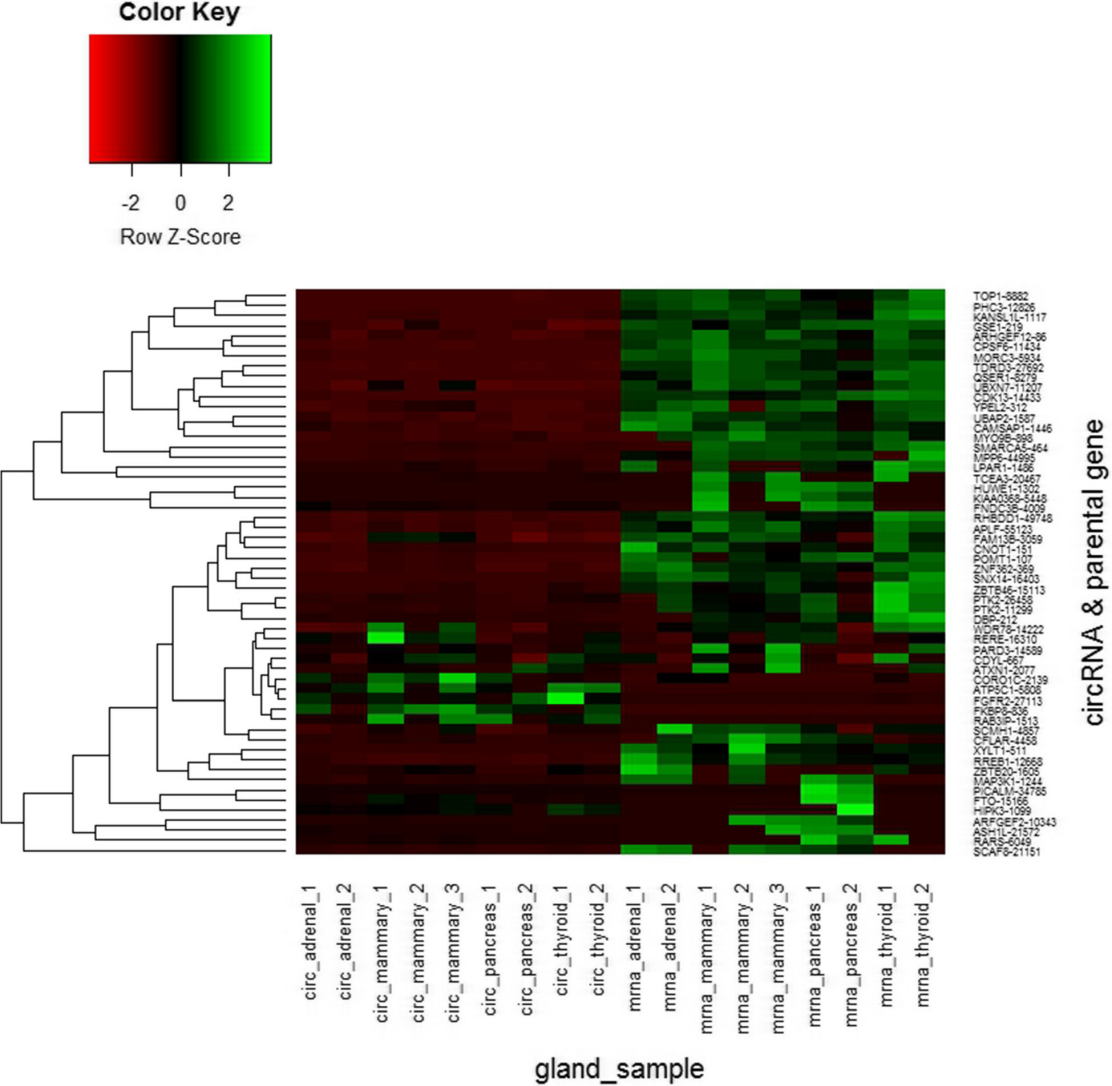
Heatmap showing the expression of the 56 common circRNAs and their parental mRNAs in gland normal tissues

**Fig. 9:**
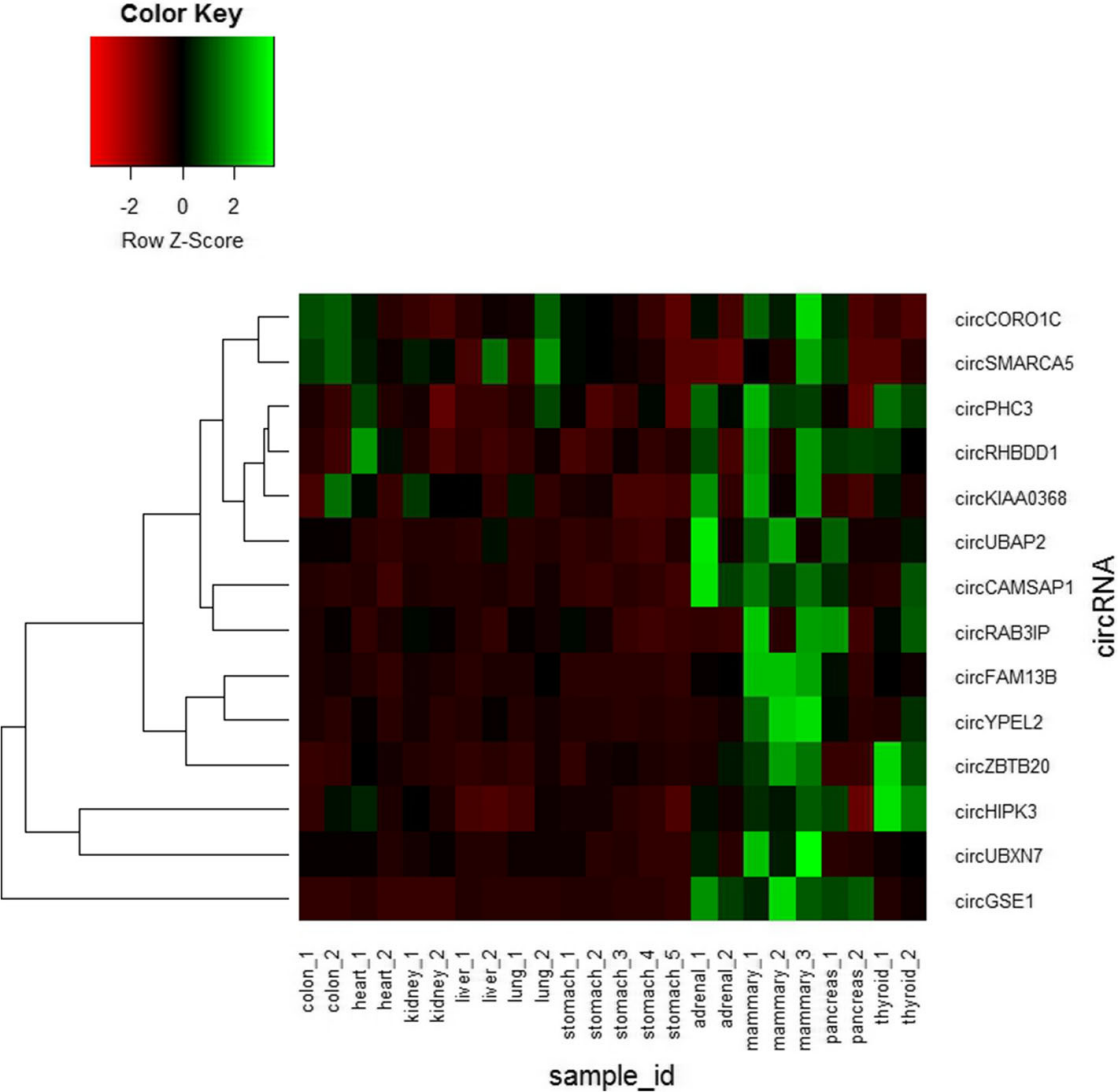
Heatmap showing the expression of the 14 shared circRNA in each sample of adult tissues and gland tissues

Furthermore, our results revealed 56 circRNAs had shared expression in different gland normal tissues. Most of circRNAs’ expression level was lower than their parental mRNAs’ expression, but six circRNAs (circFKBP8, circATP5C1, circFGFR2, circRAB3IP, circCORO1C, and circHIPK3) presented the opposite expression with their corresponding parental genes. CircFTO and circPICALM present same trend in adrenal gland, mammary gland and thyroid gland, but high expression of their parental mRNA was observed in pancreas (Fig. 8). In addition, there were 14 circRNAs identified in each sample of human adult tissues. Compared with circRNAs expression in adult tissues, these 14 circRNAs in gland tissues expressed higher than that in adult tissues (Fig. 9). Previous work has revealed that circHIPK3 can regulate cell growth by sponging multiple miRNAs [15]. Our finding also revealed that circHIPK3 expressed higher than their parental linear mRNA in each gland sample. These results might be attributed to the special function of glands in human body like secreting hormonal substances to regulate cell growth.

## Conclusions

In this work, we identified 8120 circular RNAs in six human adult normal tissues (colon, heart, kidney, liver, lung, and stomach), 25,933 in the human fetal normal tissues, and 13,374 in human gland normal tissues (adrenal gland, mammary gland, pancreas, and thyroid gland). Compared with the recent databases, 38% and 66% circRNAs in adult and fetal tissues were identified as novel. Further analysis showed that each adult tissue expressed a certain number of unique circRNAs: 441 (36.97%) in colon, 602 (50.04%) in heart, 474 (40.86%) in kidney, 668 (49.34%) in liver, 452 (36.92%) in lung, and 528 (40.80%) in stomach. The results indicated that expression of circRNAs was tissue-specific.

There were 33 circRNAs detected ubiquitously in each sample of adult normal tissues. We constructed two networks: one included these 33 circRNAs and their potential target miRNAs and the other is a circRNA-miRNA-mRNA network. These two networks indicated the potential associations between circRNAs and their target genes. The networks provided an important reference for studying the interaction of other differential expressed circRNAs and their potential targets.

The abundance of circRNAs in fetal tissues was stronger than that in adult tissues. The relative expression level of circRNAs and their parental mRNAs in colon and liver samples were higher in fetal tissue than adult tissue. The results suggested that circular RNA might have important roles during human tissue development. Future efforts should be directed to illuminate the specific molecular function of each circRNA during the development in each tissue type.

Finally, we studied the circRNA-specific expression profiles in four gland normal tissues (adrenal gland, mammary gland, pancreas, and thyroid gland). The abundance of circRNAs was stronger in gland tissues than human adult tissues. We identified 14 conserved circRNAs shared among samples of human tissues. These 14 circRNAs expressed higher in gland tissues than adult tissues. The circRNA expression level in mammary gland was higher than other gland tissues, indicating that circRNAs might have tissue-specific regulation in glands.

## Additional files

Additional file 1: Table S1. RNA-seq data information; **Table S2.** Characteristics of known circRNAs identified in human adult tissues; **Table S3.** Characteristics of novel circRNAs identified in human adult tissues; **Table S4.** Characteristics of 33 candidate circRNAs identified in human adult tissues; **Table S5.** Characteristics of 33 circRNAs parental gene expression in human adult tissues; **Table S6.** Characteristics of circRNAs identified in human fetal tissues; **Table S7.** Characteristics of circRNAs identified in human gland tissues; **Table S8.** Gene Ontology analysis of biological processes in circRNAs of only in fetal tissues. (XLSX 3123 kb)

Additional file 2: Figure S1. The top 10 circRNAs expression of each sample in six human adult normal tissues. (JPEG 161 kb)

Additional file 3: Figure S2. The biological processes and molecular function of GO enrichment in 90 mRNAs-derived genes of circRNA-miRNA-mRNA network. (JPEG 248 kb)

Additional file 4: Figure S3. The circRNAs expression and their parental mRNA expression in adult and fetal colon tissue. (JPEG 531 kb)

Additional file 5: Figure S4. The circRNAs expression and their parental mRNA expression in adult and fetal liver tissue. (JPEG 519 kb)

Additional file 6: Figure S5. The circRNAs expression and their parental mRNA expression in adult and fetal heart tissue. (JPEG 514 kb)

Additional file 7: Figure S6. The circRNAs expression and their parental mRNA expression in adult and fetal kidney tissue. (JPEG 516 kb)

Additional file 8: Figure S7. The circRNAs expression and their parental mRNA expression in adult and fetal lung tissue. (JPEG 507 kb)

Additional file 9: Figure S8. The circRNAs expression and their parental mRNA expression in adult and fetal stomach tissue. (JPEG 510 kb)

## Abbreviations

BMJ: balanced mapped junction
GO: Gene Ontology
IGV: Integrative Genomics Viewer
UMJ: unbalanced mapped junction
