# Supplementary figures and images for: Circular RNA expression profiles and features in human tissues: a study using RNA-seq data
Source: BMC Genomics. 2017 Oct 3;18(Suppl 6):680. doi: 10.1186/s12864-017-4029-3 (PMC5629547; doi:10.1186/s12864-017-4029-3)

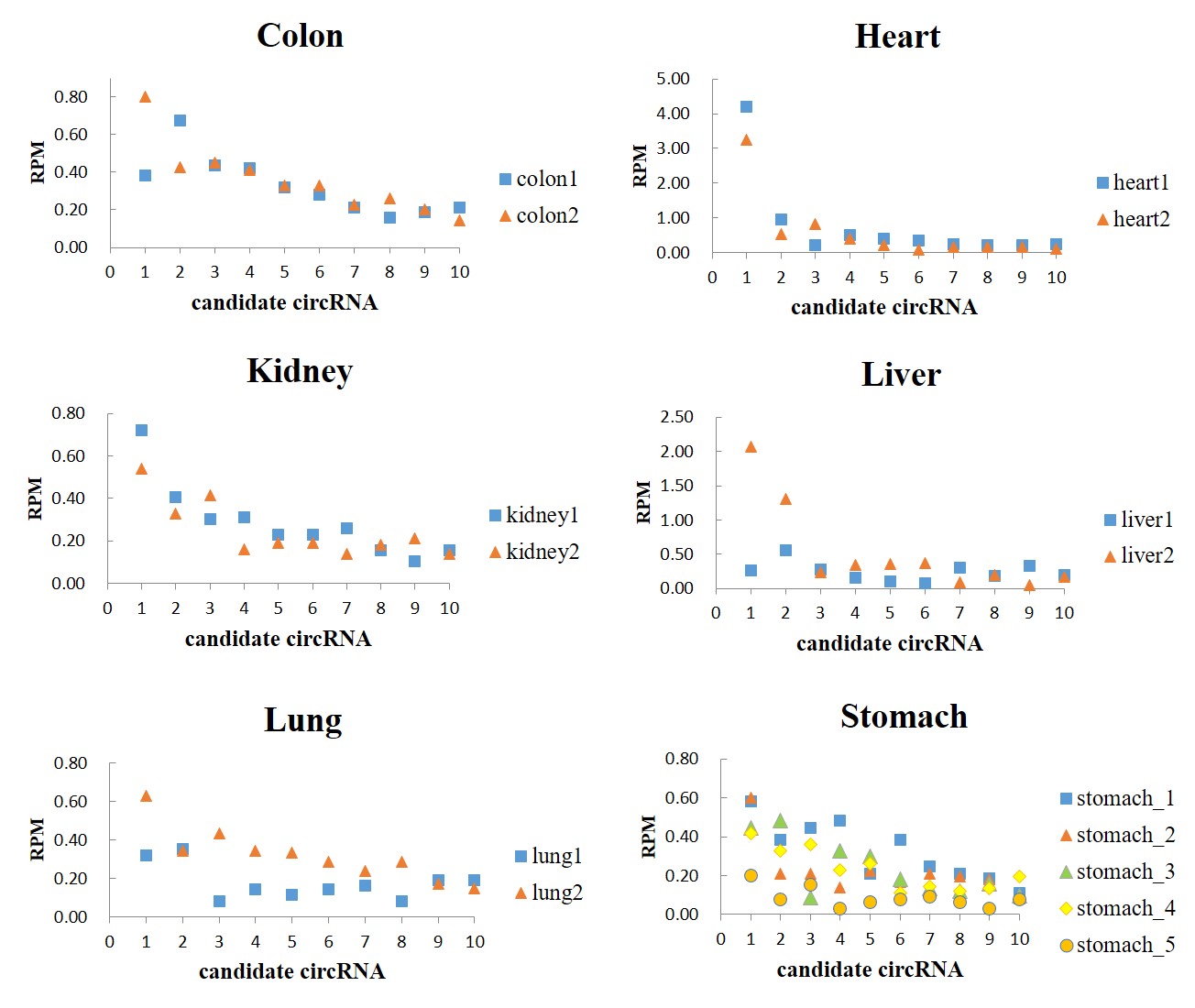

Supplement: Supplementary file 2 — The top 10 circRNAs expression of each sample in six human adult normal tissues. (JPEG 161 kb) [file 12864_2017_4029_MOESM2_ESM.jpg]

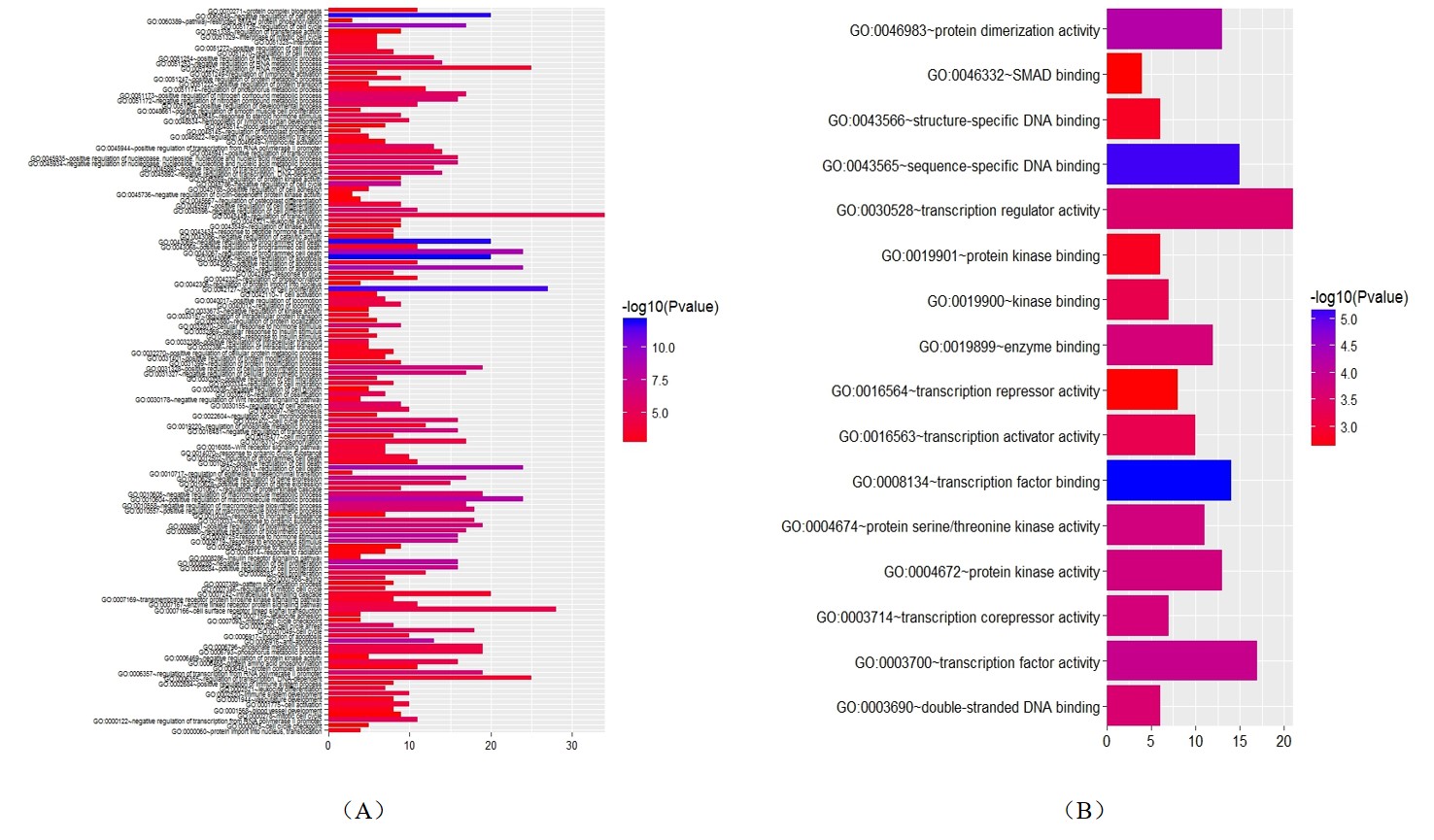

Supplement: Supplementary file 3 — The biological processes and molecular function of GO enrichment in 90 mRNAs-derived genes of circRNA-miRNA-mRNA network. (JPEG 248 kb) [file 12864_2017_4029_MOESM3_ESM.jpg]

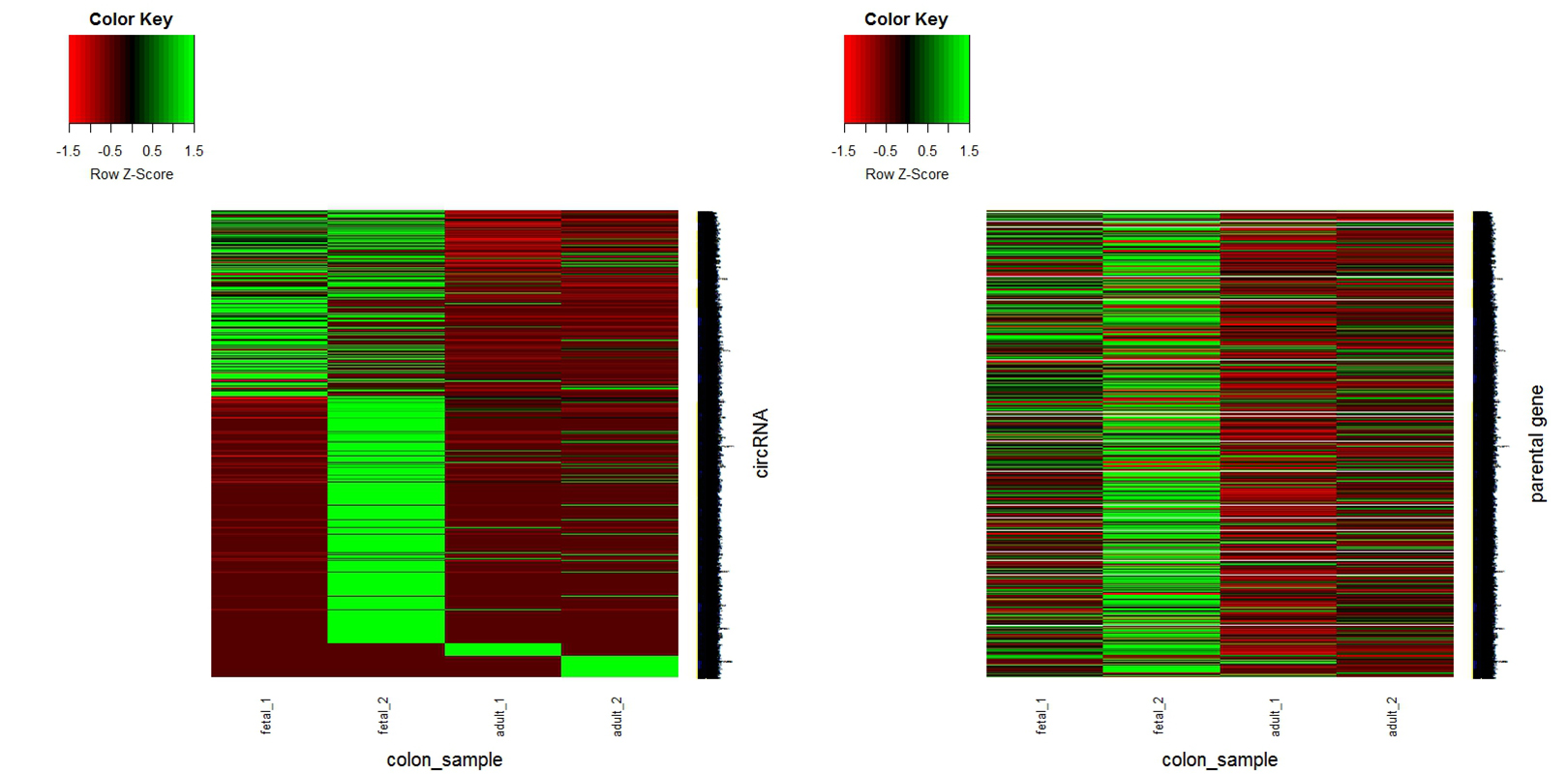

Supplement: Supplementary file 4 — The circRNAs expression and their parental mRNA expression in adult and fetal colon tissue. (JPEG 531 kb) [file 12864_2017_4029_MOESM4_ESM.jpg]

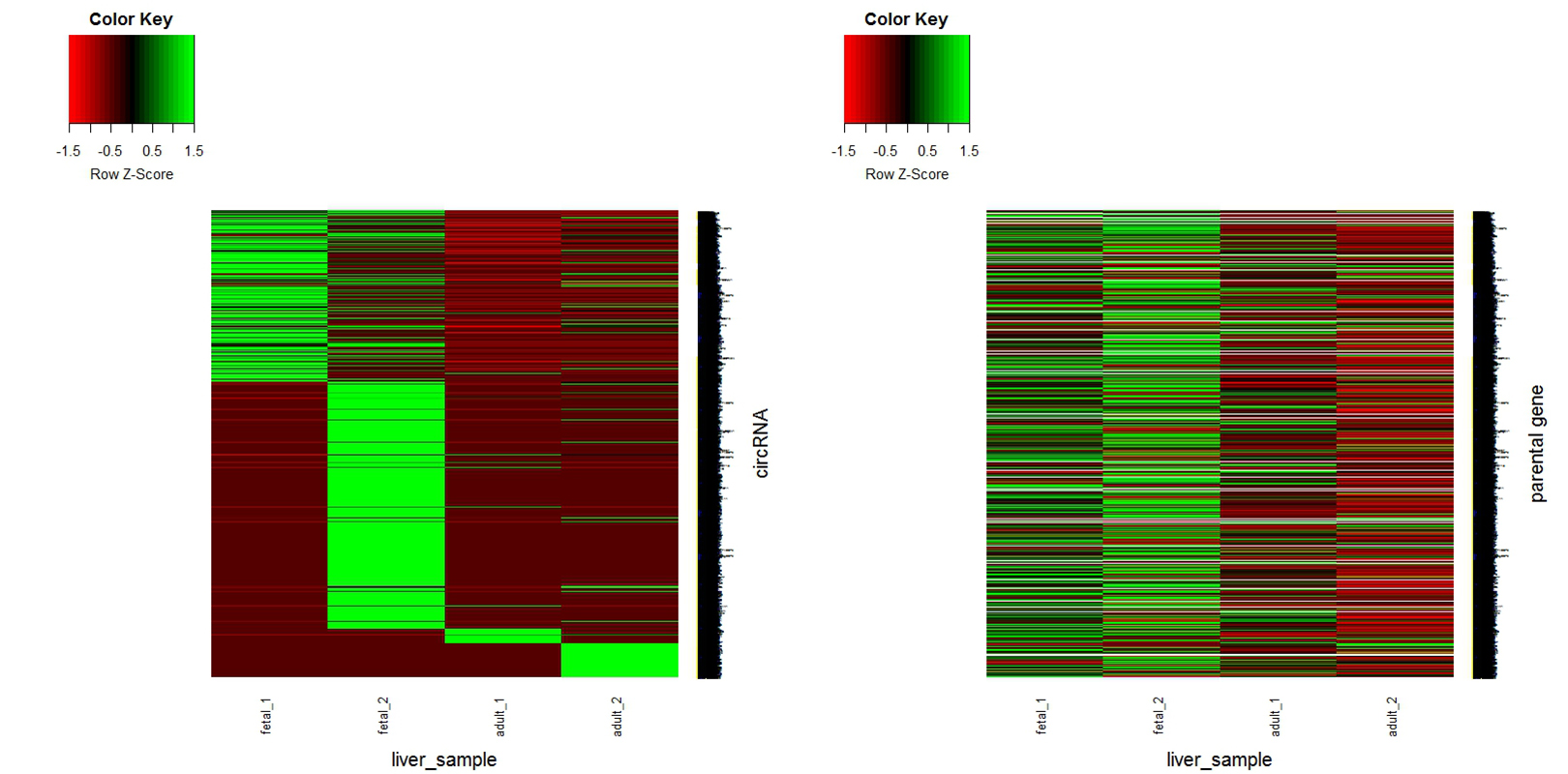

Supplement: Supplementary file 5 — The circRNAs expression and their parental mRNA expression in adult and fetal liver tissue. (JPEG 519 kb) [file 12864_2017_4029_MOESM5_ESM.jpg]

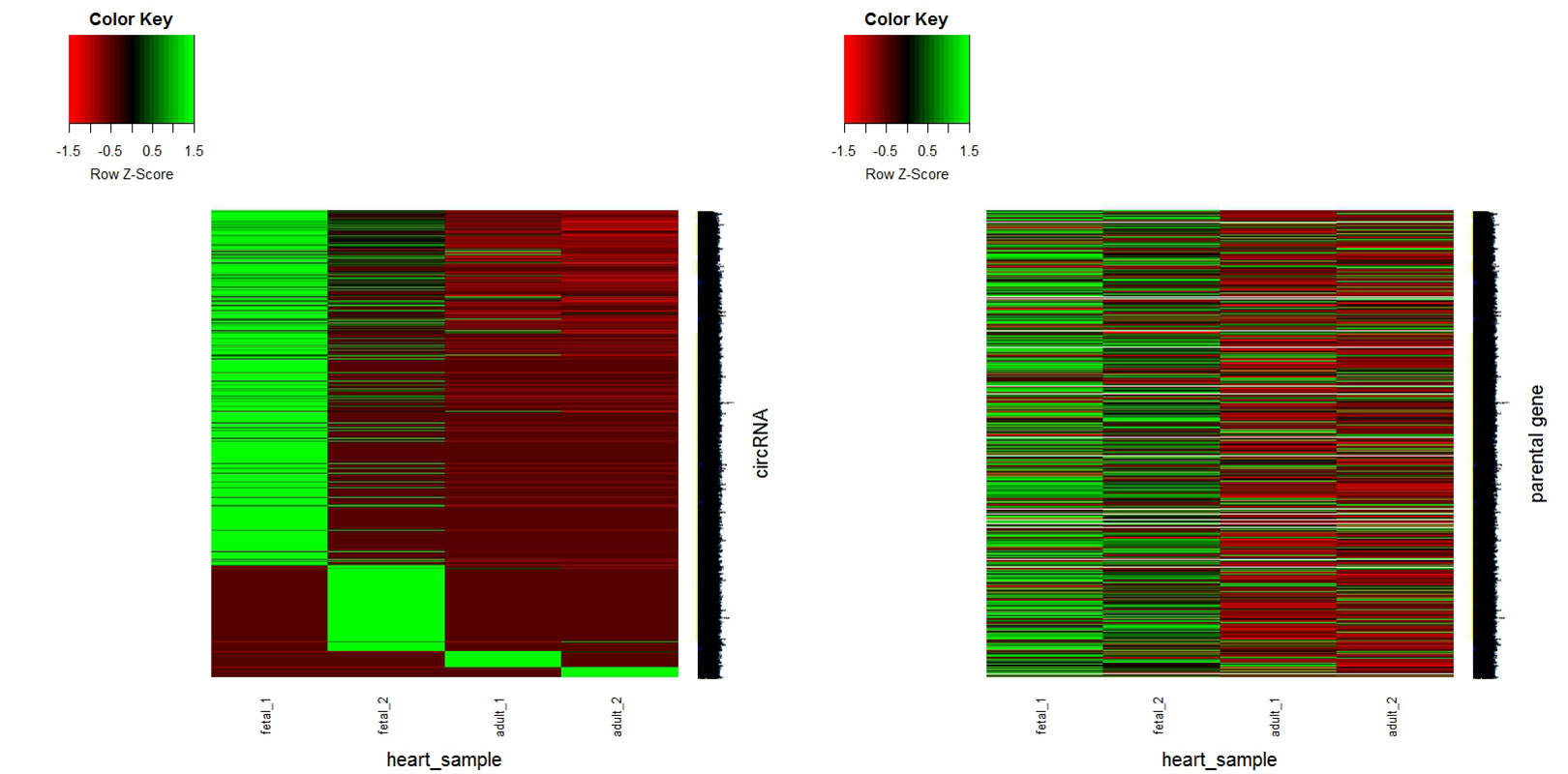

Supplement: Supplementary file 6 — The circRNAs expression and their parental mRNA expression in adult and fetal heart tissue. (JPEG 514 kb) [file 12864_2017_4029_MOESM6_ESM.jpg]

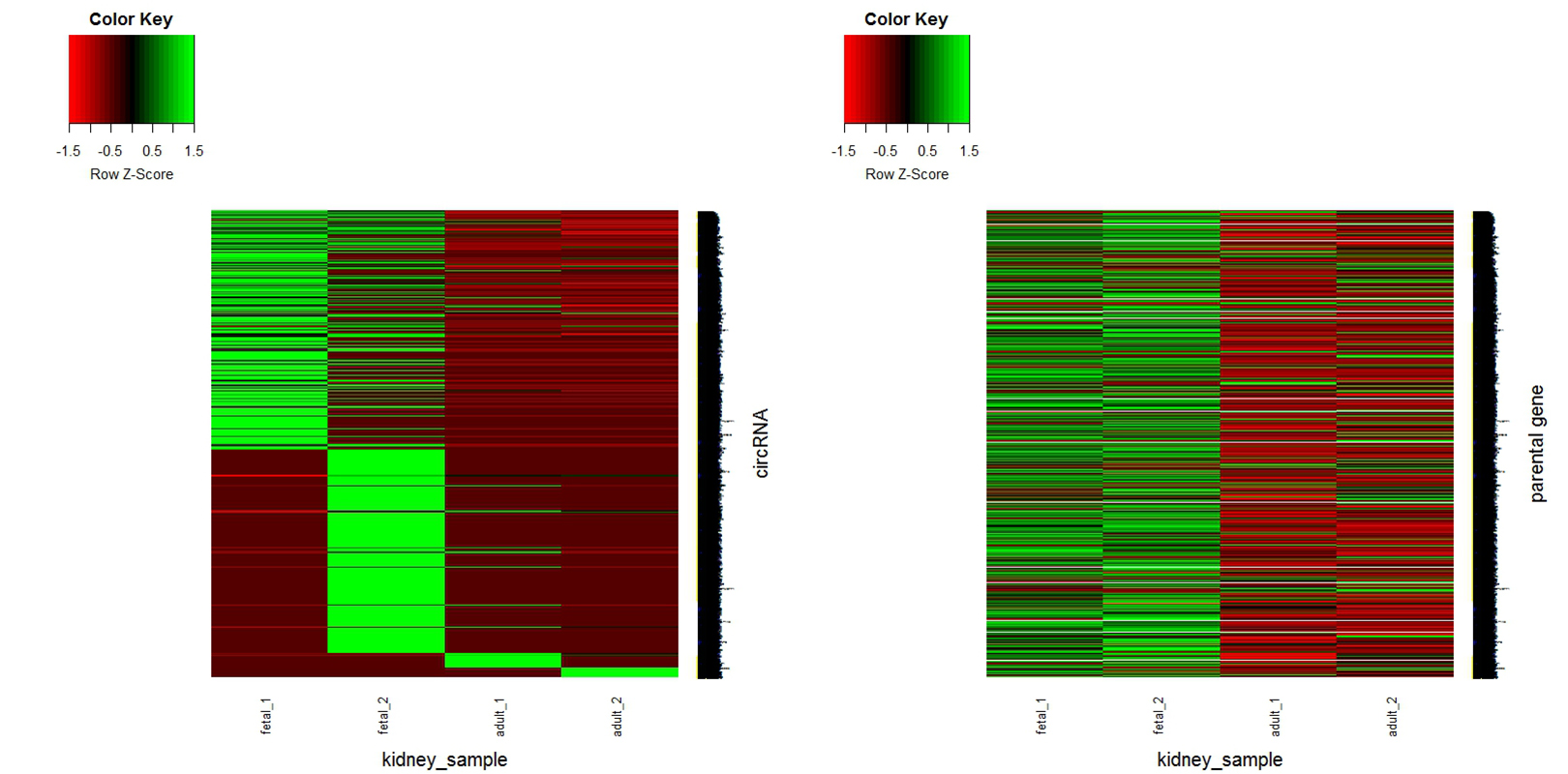

Supplement: Supplementary file 7 — The circRNAs expression and their parental mRNA expression in adult and fetal kidney tissue. (JPEG 516 kb) [file 12864_2017_4029_MOESM7_ESM.jpg]

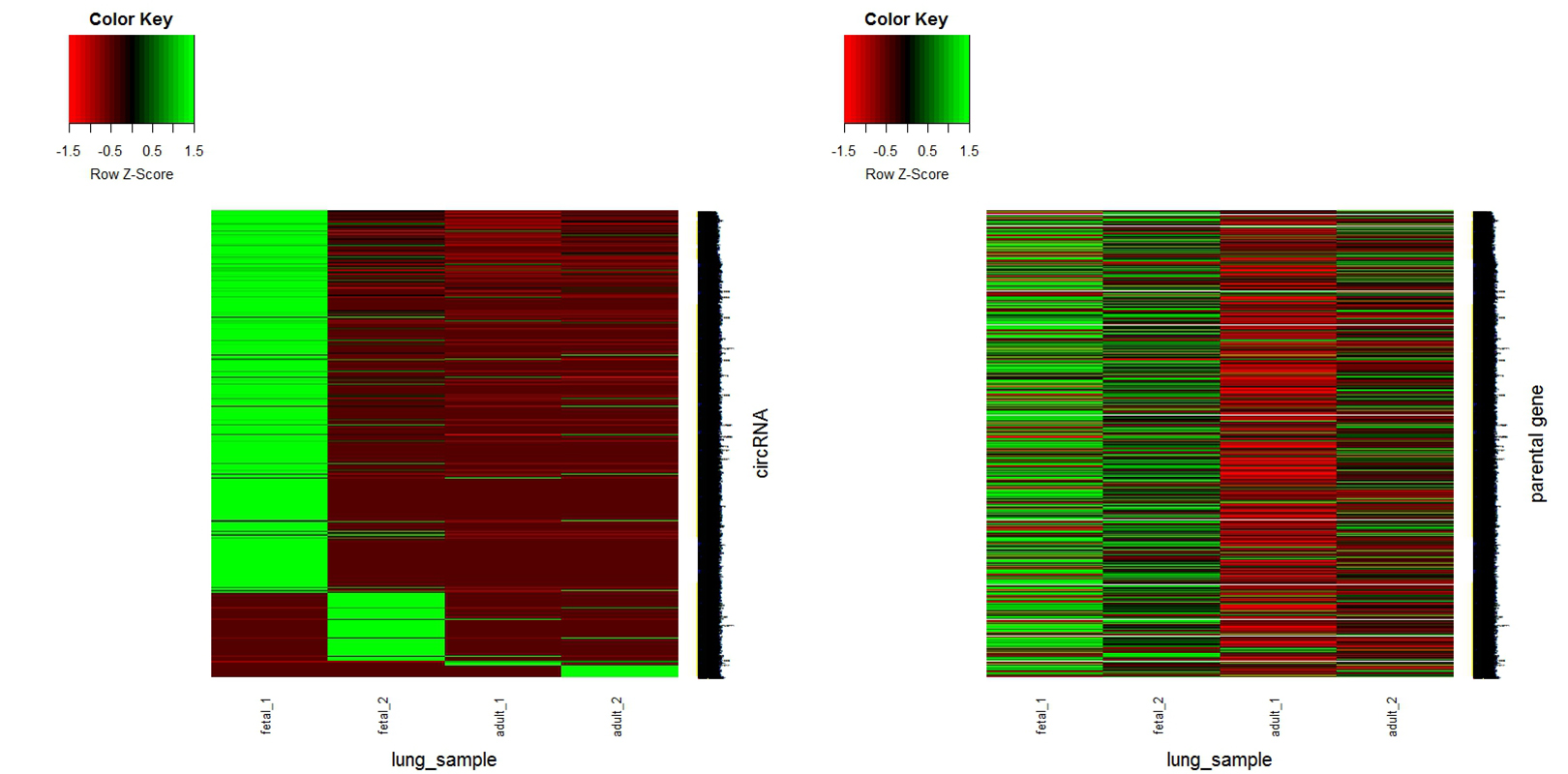

Supplement: Supplementary file 8 — The circRNAs expression and their parental mRNA expression in adult and fetal lung tissue. (JPEG 507 kb) [file 12864_2017_4029_MOESM8_ESM.jpg]

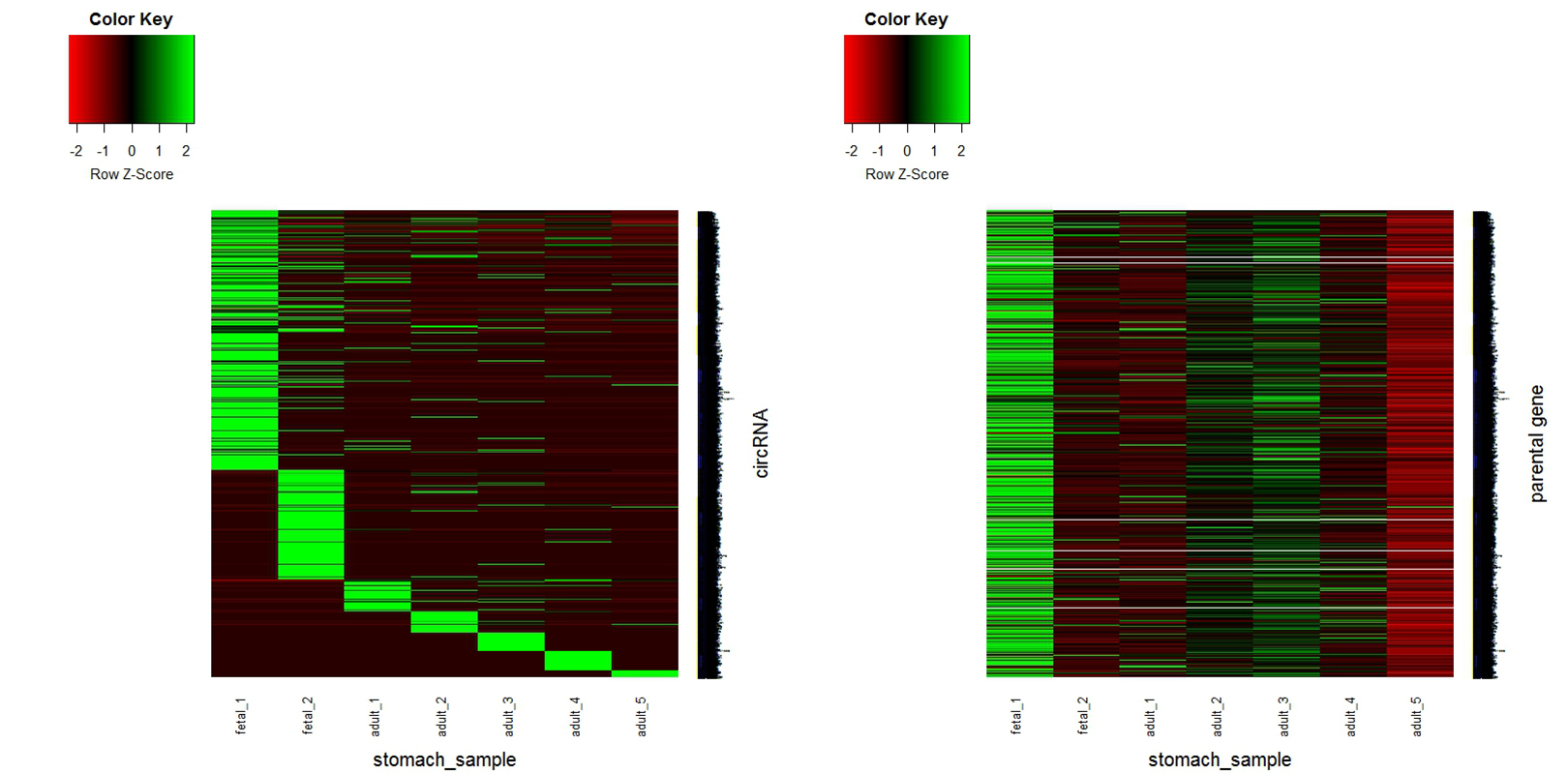

Supplement: Supplementary file 9 — The circRNAs expression and their parental mRNA expression in adult and fetal stomach tissue. (JPEG 510 kb) [file 12864_2017_4029_MOESM9_ESM.jpg]
